# Methodological adaptations for applying the Harmonised Cognitive Assessment Protocol diagnostic algorithm in the Northern Ireland Cohort for the Longitudinal Study of Ageing (NICOLA-HCAP)

**DOI:** 10.1186/s13104-026-07847-x

**Published:** 2026-05-02

**Authors:** Nicola Ann Ward, Calum Marr, Stanley Simoes, Leeanne O’Hara, Michael McAlinden, Charlotte Sterling, Claire Potter, Frank Kee, Peter Passmore, Angie Scott, David R. Weir, Bernadette McGuinness

**Affiliations:** 1 Centre for Public Health, Queen’s University Belfast, Belfast, UK; 2 Northern Ireland Clinical Research Network, Belfast Health and Social Care Trust, Belfast, UK; 3 University of Michigan, Ann Arbor, MI USA

**Keywords:** NICOLA, Harmonised Cognitive Assessment Protocol, Population ageing, Dementia, Mild cognitive impairment, Prevalence, Cross-national harmonization, Classification algorithm, Normative sample, Factor analysis

## Abstract

**Objective:**

Nationally representative data are essential for understanding the causes, consequences, and costs of dementia and mild cognitive impairment (MCI) and for informing policy and care planning. This study aimed to describe methodological considerations in applying the Health and Retirement Study Harmonised Cognitive Assessment Protocol (HRS-HCAP) cognitive domain structure and diagnostic algorithm to the Northern Ireland Cohort for the Longitudinal Study of Ageing (NICOLA-HCAP), and to generate harmonised estimates of the prevalence of dementia and mild cognitive impairment (MCI) in a nationally representative sample.

**Results:**

A total of 1,037 participants aged ≥ 65 years completed NICOLA-HCAP. Five cognitive domains were identified, all loading onto a second-order general cognitive performance factor: orientation (0.903), memory (0.855), executive function (0.893), language-fluency (0.962), and visuospatial ability (0.812). Model fit was acceptable (SRMR = 0.065; RMSEA = 0.047; CFI = 0.916; TLI = 0.906). Following classification, 6.2% of participants were classified with dementia and 15.8% as MCI. Methodological modifications addressed software differences, normative sample derivation, and cohort-specific adjustments. These findings provide preliminary support for HCAP as a framework for producing harmonised estimates of cognitive status. NICOLA-HCAP will facilitate future investigation of modifiable risk factors for dementia in community-dwelling older adults. Validation studies are required to determine whether resulting classifications are fit for purpose.

**Supplementary Information:**

The online version contains supplementary material available at 10.1186/s13104-026-07847-x.

## Introduction

Dementia is a global public health crisis [1], characterised by progressive cognitive decline, loss of independence and impaired activities of daily living (ADLs) [2]. Dementia is often preceded by mild cognitive impairment (MCI), progressing at 8–15% annually [3]. It imposes a substantial burden on individuals, caregivers, and healthcare systems, costing nearly £1 billion per year in Northern Ireland (NI) [4]. Current prevalence estimates may be unrepresentative due to underdiagnosis in primary care [5, 6], lengthy diagnostic pathways [7] and exclusion of vulnerable populations from datasets [8], highlighting the need for representative population-based data to inform care planning and policy in NI.

The Harmonised Cognitive Assessment Protocol (HCAP), originally implemented in the US Health and Retirement Study (HRS), was adopted in the NI Cohort for the Longitudinal Study of Ageing (NICOLA) [9]. NICOLA-HCAP intentionally mirrors sister studies: the Irish Longitudinal Study on Ageing (TILDA) and the English Longitudinal Study of Ageing (ELSA) [10]. NICOLA-HCAP is part of the global HCAP network [10–15] aiming to generate internationally comparable dementia prevalence data using a flexible instrument, “HCAP” for population-level analyses, not intended for clinical diagnosis. To achieve this, protocols across studies incorporate adaptations to test items, administration, and scoring to accommodate linguistic, cultural, and educational differences [16].

The suitability of HCAP across diverse populations remains uncertain, particularly in regions with distinctive sociocultural profiles. In NI, cognitive ageing and resilience may be influenced by the legacy of prolonged political conflict (“The Troubles” 1969–1999) and long term health, behaviour and socioeconomic consequences [17]. NICOLA data showed conflict-related events during the Troubles were associated with better later-life memory, moderated by social activity engagement, potentially reflecting cohort, survivor, or recall mechanisms [18]. More recent findings showed that those with significant post-traumatic stress disorder had worse cognitive performance, with a strong association between depression and health behaviours [17]. NI also has some of the highest levels of deprivation and depression in the UK [19], and a mixed rural–urban population [20] compared with TILDA [21] and ELSA [10]. ELSA-HCAP found that the psychometric properties of identical tests can vary across cultural contexts [22]. Other HCAP studies have reported challenges arising from heterogeneity across countries and from missing data when applying this methodology to different cohorts [23].

Following cross-national harmonisation guidelines [20], this work aimed to replicate the HRS-HCAP protocols [24, 25] within the NI context and detail the methodological adaptations needed to establish cognitive status estimates in NICOLA-HCAP.

### Objectives

1. Evaluate the five-domain cognitive model in NICOLA-HCAP using confirmatory factor analysis (CFA).
2. Standardise domain-specific cognitive scores against an adjusted robust normative sample.
3. Apply the HRS-HCAP diagnostic algorithm thresholds to classify cognitive status in NICOLA-HCAP.

## Main text

### Study population and design

NICOLA Wave 1 (W1) began in 2013 with 8,478 individuals aged 50+ [9, 26]. W2 (2017–2019) saw 6,852 participants (81%) complete follow-up interviews, and W3 began in 2024. The NICOLA-HCAP sub-study (2022–2023) recruited a random sample of NICOLA W2 participants aged 65 + living in the community [27, 28]. Assessments included a computer-assisted personal interview (CAPI) with 19 neuropsychological tests and a family or friend “informant” interview. The main study received ethical approval from Queen’s University Belfast Faculty of Medicine, Health and Life Sciences Research Ethics Committee [29]. Participants gave written informed consent or proxies did. The NICOLA-HCAP study followed the Declaration of Helsinki.

### Measures

The rationale for including cognitive measures was previously described [27]. Briefly, the NICOLA-HCAP adopted the HRS-HCAP cognitive battery [25], which prioritises assessment across multiple cognitive domains using established neuropsychological measures of memory[language-fluency, executive function visuospatial ability, and orientation. The battery comprised tasks including word list learning and recall, story memory, semantic fluency, attention and speed assessments, reasoning, and constructional praxis to support research classification of MCI and dementia, while enabling harmonisation with international HCAP studies and longitudinal evaluation of cognitive decline. Items requiring cultural adaptation (e.g., naming the President) were retained using equivalent formats suitable for the UK context (e.g., naming the Prime Minister). To assess functional change, which is embedded in diagnostic criteria for dementia [30], informant interviews incorporated validated measures [27, 28] to capture perceived decline in cognition, everyday functioning, and daily activities.

### Statistical analysis

**Fig. 1:**
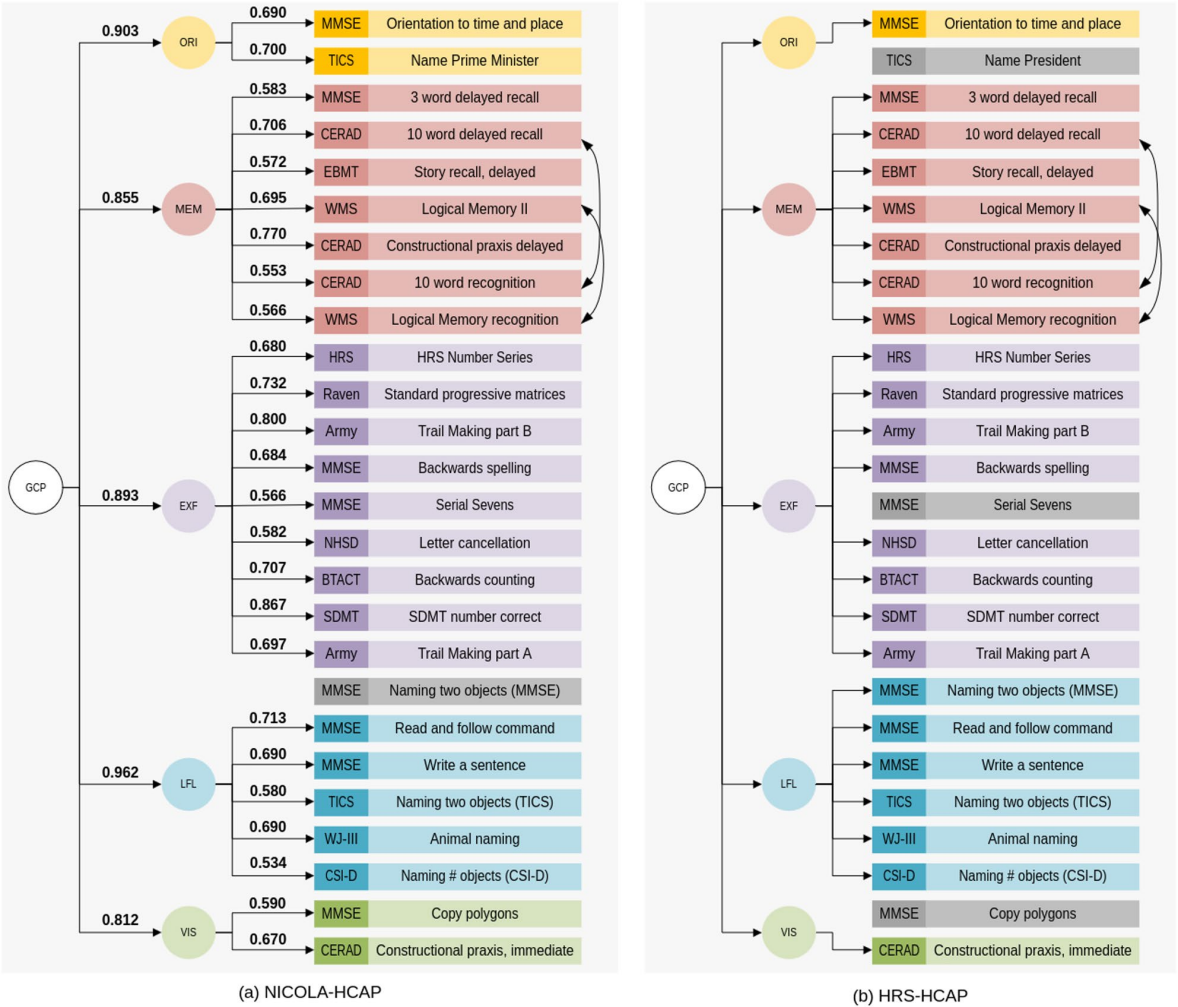
Second-order factor models of GCP with five latent cognitive domains in NICOLA-HCAP versus HRS-HCAP. Panel (**A**) summary structure in NICOLA-HCAP; panel (**B**) corresponding structure from HRS-HCAP. Variables in grey were not used in the final model. Reported factor loadings represent the mean across the nine successfully estimated models (1 failed to converge). *ORI* orientation; *LFL* language and fluency; *VIS* visuospatial; *MEM* memory; *EXF* executive functioning; *GCP* general cognitive performance

Analyses consisted of four stages: [1] data cleaning in preparation for CFA, [2] application of prespecified HRS-HCAP domains to NICOLA-HCAP via CFA and estimation of factor scores, [3] identification and standardisation of a robust normative sample, adjusting for sociodemographic characteristics, and [4] dementia status classification using the HRS-HCAP algorithm (refer to Additional Files 1: Fig. 1).

Due to software availability, all analyses were conducted in R (version 4.5.2; R Core Team 2024) rather than Mplus. Adaptations were made to the original protocol code [31]. CFA models used the *lavaan* package [32]. Missing data were handled with Multiple Imputation by Chained Equations (MICE) from the *mice* package [33], and robust normative samples were adjusted using restricted cubic splines in the *rms* package [34].

### Data cleaning

Cognitive variables were grouped into domains per HRS-HCAP framework. Data was cleaned and harmonised in line with HRS sister studies. Full data handling procedures are described in Additional Files 2.

### CFA

#### Modelling

In HRS-HCAP, CFA reduced eight hypothesised cognitive domains to five: orientation (ORI), memory (MEM), executive functioning (EXF), language/fluency (LFL), and visuospatial ability (VIS) using WLSMV (Weighted Least Squares Mean and Variance adjusted). Final factor scores were estimated using MLR (maximum likelihood with robust standard errors) (see [24]).

In NICOLA-HCAP, this approach was replicated in R but WLSMV was unsuitable due to its reliance on listwise deletion for missing data when using categorical indicators [35, 36]. This can bias results if data are not missing completely at random (MCAR), particularly when missingness may be informative (e.g., signalling cognitive decline). In contrast, estimators such as MLR handle missing data more flexibly under the missing at random assumption but all variables must be treated as continuous. For NICOLA-HCAP, the continuous data assumption caused convergence issues in R. We used MICE to generate 10 imputed datasets before CFA, as ML and MICE produce similar results [37, 38]. This maintains the categorical structure of several indicators while minimising data loss and avoids choosing between inappropriate estimators and suboptimal missing data strategies. Model fit was assessed using Comparative Fit Index (CFI) and Tucker–Lewis Index (TLI) values ≥ 0.95, Root Mean Squared Error of Approximation (RMSEA) values ≤ 0.06, and Standardised Root Mean Square Residual (SRMR) values ≤ 0.08 [39]. Factor loadings were interpreted using previously published guidelines, where values ≥ 0.50 were considered meaningful and ≥ 0.70 considered strong [40, 41].

## Results

NICOLA-HCAP successfully replicated the five-domain model (ORI, MEM, LFL, EXF, VIS), Fig. 1. Like HRS, immediate memory indicators were excluded due to high correlations with delayed and recognition memory measures, which remained in the MEM domain. Unlike HRS-HCAP, which dropped two indicators (Fig. 1B), specifically naming the president (from ORI) and copying polygons (from VIS), leaving each domain with a single observed score, NICOLA-HCAP retained both variables to preserve indicator variability and measurement reliability. The MMSE naming score within the LFL domain was dropped, as 99.6% of participants achieved the maximum score (see Fig. 1A).

Factor scores were calculated for all 1,037 participants. A detailed missingness breakdown is in Additional Files 3: Table 1A, 1B. The final CFA model demonstrated acceptable fit: CFI = 0.916, TLI = 0.906, RMSEA = 0.047, and SRMR = 0.065. All first-order cognitive domains loaded strongly onto a second-order general cognitive performance (GCP) factor: ORI (0.903), MEM (0.855), EXE (0.893), LFL (0.962), and VIS (0.812). The full structural equation model, including latent factors and standardised loadings means, Fig. 1A.

### Normative sample

To establish expected cognitive performance levels for cognitively healthy participants, a robust normative sample was constructed based on demographic characteristics. HRS-HCAP leveraged longitudinal Medicare claims data to create a normative sample by excluding those with cognitive impairment, stroke, Parkinson’s disease, related dementia and nursing home residents. In contrast, NICOLA-HCAP applied exclusion criteria based on cognitive screeners, functional limitations in ADLs, and informant-reported symptom progression, harmonised with other sister longitudinal ageing studies, which reported similar issues with normative sample creation [23, 42–44]. The robust normative sample was predefined by the HCAP team, as the final cognitive classification is sensitive to the composition of the normative group. The number of participants removed for each criterion and the rationale for their inclusion are shown in Additional Files 4: Table 1.

The final normative group (*n* = 477; 46% of the HCAP sample) was comparable to the full cohort in age (mean=72years, range: 65–91) and sex (56% female). There was a significant difference in educational level between the normative and full samples, i.e., 17% versus 10% with primary education or below (*P* < 0.001). Domain factor scores were rank-normalised and adjusted for age, sex, and education, following HRS-HCAP, and, additionally, for risk of depression using the CES-D-*short-form* [45] based on evidence that depressive symptoms are associated with lower cognitive test performance, regardless of neurodegeneration [46–48]. It is worth noting that NICOLA-HCAP participants reported higher depressive symptoms (~ 28%) than TILDA-HCAP (~ 18%) and ELSA-HCAP(-3% ) [10, 21, 28]. This aligns with memory clinic practice, where mood screening is standard to distinguish depression-related cognitive impairment (“pseudodementia”) from dementia [49].

The rank-normalised scores of the normative sample were regressed on the above four factors to yield expected scores for all combinations of age, sex, education, and depression. Full standardisation procedures are in Additional files 4. Since adjusting for depression deviates from HRS-HCAP, domain scores adjusted only for age, sex, and education, as well as HCAP-derived cognitive status, will also be made available for harmonisation purposes via the NICOLA Data Access Committee. Information on data access is available on the NICOLA study website (https://www.qub.ac.uk/sites/NICOLA/).

### Algorithm

The HRS-HCAP algorithm was replicated to classify participants as cognitively normal, MCI, or dementia, using T-scores ≤ 35 (equates to 1.5 SD below the mean) and self- or informant-reported concerns. Dementia required impairment in ≥ 2 domains plus informant-rated decline, MCI required ≥ 1 impaired domain with informant or self-rated decline, and all others were classified as normal.

### Output and classification

**Fig. 2:**
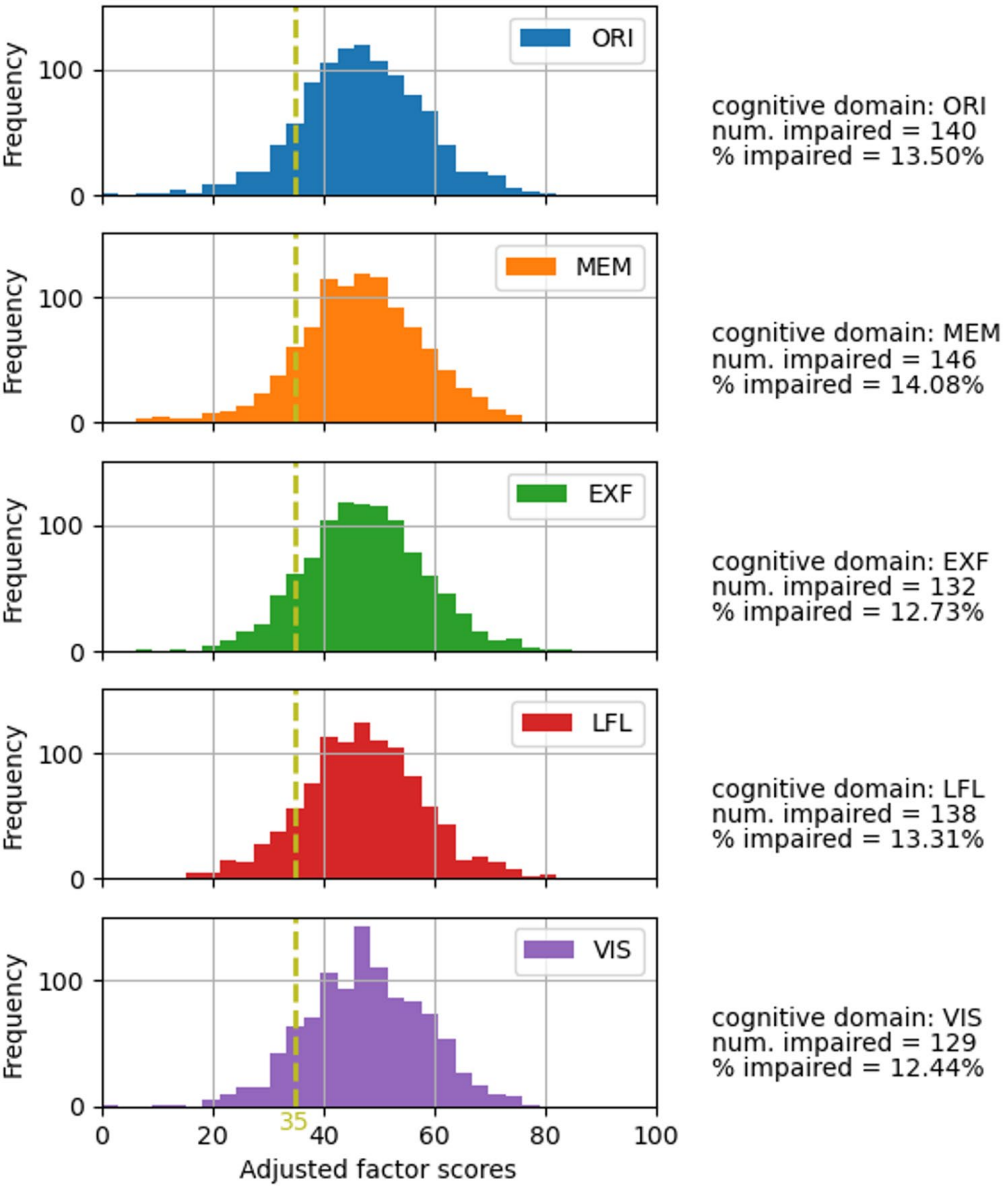
Histograms of adjusted factor scores for each cognitive domain. Factor scores (T scores) ≤ 35 indicate cognitive impairment. *ORI* orientation; *LFL* language and fluency; *VIS* visuospatial; *MEM* memory; *EXF* executive functioning; *GCP* general cognitive performance

**Fig. 3:**
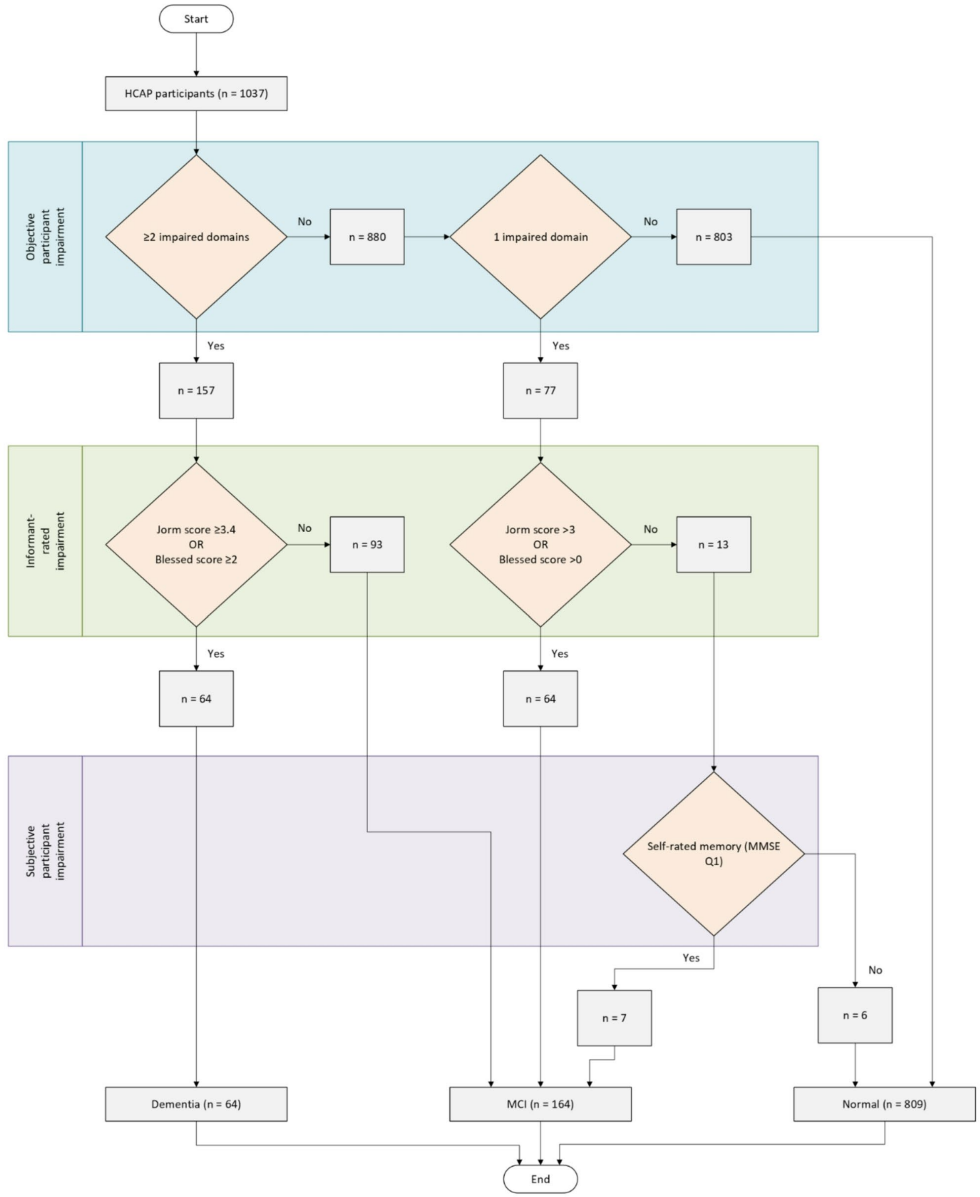
Flow diagram showing the HRS-HCAP diagnostic algorithm for classification of Dementia, MCI or Normal Cognition. *MCI* Mild cognitive impairment; *JORM score* JORM informant Questionnaire on Cognitive Decline in the Elderly score;* Blessed score* Dementia Rating Scale Part 1

Following adjustment of factor scores (Fig. 2), cognitive impairment was highest in MEM (*n* = 146;14.1%) and lowest in VIS (*n* = 129;12.4%). Figure 3 illustrates the breakdown of the diagnostic algorithm. 157 individuals (15.1%) showed impairment in ≥ 2 domains, with almost 40.8% (*n* = 64) having informant-reported impairment. Among those with single-domain impairment (*n* = 77;7.4%), over 83.1% (*n* = 64) had informant-rated concerns. Of those without, over half (*n* = 7;53.8%) reported memory concerns themselves. Following classification, 64 NICOLA-HCAP participants (6.2%) were identified as having dementia, and 164 (15.8%) as having MCI.

Additional Files 5: Fig. 1 illustrates the distribution of adjusted GCP scores across diagnostic groups. Overall, there were 72 cases where informant-rated impairment contributed substantially to the diagnostic process, and GCP alone would not have been able to distinguish between normal cognition, MCI and dementia.

## Discussion

The HRS-HCAP methodology was applied to the NICOLA-HCAP sample, with adaptations for demographic differences, model performance, missing data patterns, and software availability. Regardless, the model demonstrated reasonable fit, with factor scores comparable to those of HRS, supporting the cross-cohort applicability and flexibility of HCAP [24].

We report prevalence estimates for community-dwelling adults from a nationally representative sample [26]. Direct estimates with HRS are currently not possible because, unlike HRS, NICOLA W1 and W2 did not recruit individuals from care home settings; therefore, final prevalence estimates for this subgroup are zero. Participants’ transition into residential care is being monitored in W3. This methodology aligns with other longitudinal ageing studies that started with community-based samples, including ELSA, CHARLS, and HRS.

The 6.2% dementia prevalence in NICOLA-HCAP exceeds UK primary care estimates (~ 3.6–4.8% among older adults [50]), likely because primary care estimates only include formally diagnosed cases. It is also well known that dementia is underdiagnosed in community settings, especially in early stages. Consequently, estimates from HCAP-based studies are more likely to reflect true underlying population estimates [51, 52]. SHARE-HCAP studies also estimate dementia prevalence between 6 and 10% in age-matched adults across countries [23].

Strengths include the creation of a normative sample that avoided excessive restriction and ensured representation of older individuals, as well as the inclusion of informant data, which contributed to diagnostic classification. The pattern of GCP within the community-dwelling sample underscores the importance of the informant’s perspective in diagnosis, as a diagnosis, based solely on GCP, fails to capture overall cognitive status.

## Limitations

Dementia prevalence is substantially higher in institutionalised populations [53]. The absence of care homes in the current study may therefore contribute to an underestimation of the overall population burden in NI, suggesting that the true prevalence is likely higher than reported Cognitively impaired individuals were also excluded in W1, potentially biasing the baseline sample. Furthermore, the 7-year follow-up may not have been sufficient to capture new dementia cases. The preclinical phase of dementia, can begin 10–20 years prior to diagnosis, while the prodromal stage (often identified as MCI) typically lasts 3–7 years before progressing to dementia. However, our sister longitudinal studies have found this timeframe adequate for detecting incident dementia (HRS W15; ELSA W11).

The algorithm doesn’t explicitly handle discordant cases where informant-reported functional decline occurs without objective cognitive impairment. Currently, individuals without cognitive domain impairment are classified as normal despite informant reports, possibly missing early or functional decline not detected by cognitive tests.

Respondent burden is a limitation. The lengthy cognitive assessment battery might have caused fatigue, affecting results or causing missing data, as documented in other large ageing studies [23]. Finally, it is important to acknowledge NICOLA-HCAP is currently a cross-sectional study, which limits the assessment of cognitive decline over time. Pending funding, follow-up waves, including HCAP-2, are planned.

## Conclusion

Ongoing and future analyses will use these factor scores to investigate a range of modifiable sociodemographic, lifestyle, behavioural, and clinical risk factors associated with cognitive impairment. A clinical validation study is currently underway to evaluate the accuracy of the algorithmic classification in a memory clinic setting [54]. In conclusion, the NICOLA-HCAP dataset is a valuable resource for future research, offering comprehensive neurocognitive data.

## Supplementary Information

Below is the link to the electronic supplementary material.

Supplementary Material 1

## Data Availability

The datasets generated and/or analysed during the current study are available via the NICOLA Data Access Committee (DAC) at [nicola-research@qub.ac.uk](mailto: nicola-research@qub.ac.uk) or through external repositories, including the Dementias Platform UK (DPUK) [https://www.dementiasplatform.uk/research-hub/data-portal](https:/www.dementiasplatform.uk/research-hub/data-portal) and the Global Gateway to Aging [https://g2aging.org/home](https:/g2aging.org/home) . The NICOLA DAC can also provide code upon application approval.

## Abbreviations

ADL: Activities of daily living
IADL: Instrumental activities of daily living
CAPI: Computer-assisted personal interview
CFA: Confirmatory Factor Analysis
CFI: Comparative fit index
DAC: Data access committee
DPU: Dementias Platform UK
ELSA: English Longitudinal Study of Ageing
EXE: Executive function
HCAP: Harmonized Cognitive Assessment Protocol
HRS: Health and Retirement Study
GCP: General Cognitive Performance
GGA: Gateway to Global Aging Data platform
LFL: Language/ Fluency
MAR: Missing at random
MCI: Mild Cognitive Impairment
MEM: Memory
MMSE: Mini-Mental State Examination
NI: Northern Ireland
NICOLA: Northern Ireland Cohort for the Longitudinal Study of Ageing
ORI: Orientation
RMSEA: Root mean square error of approximation
SRMR: Standardized Root Mean Square Residual
TILDA: The Irish Longitudinal Study on Ageing
TLI: Tucker-Lewis index
VIS: Visuospatial
W: Waves
WLSMV: Weighted Least Squares Mean and Variance adjusted

## References

[1] (WHO) WHO. Global action plan on the public health response to dementia 2017–2025. 2025. https://www.who.int/news-room/fact-sheets/detail/dementia.

[2] Organization GWH. Clinical descriptions and diagnostic requirements for ICD-11 mental, behavioural and neurodevelopmental disorders2024. 2 p.

[3] Petersen RC. Mild Cognitive Impairment. Continuum (Minneap Minn). 2016;22(2 Dementia):404–18. 27042901 10.1212/CON.0000000000000313 PMC5390929

[4] Society As. Alzheimer’s Society briefing—Northern Ireland budget 2023/24. In: Martin Reilly NIONI, editor. 2024.

[5] Bradford A, Kunik ME, Schulz P, Williams SP, Singh H. Missed and delayed diagnosis of dementia in primary care: prevalence and contributing factors. Alzheimer Dis Assoc Disorders. 2009;23(4):306–314 . 10.1097/WAD.0b013e3181a6bebc PMC2787842 19568149

[6] Koch T, Iliffe S, the E-EDp. Rapid appraisal of barriers to the diagnosis and management of patients with dementia in primary care: a systematic review. BMC Fam Pract. 2010;11(1):52. 20594302 10.1186/1471-2296-11-52 PMC2909966

[7] Manthorpe J, Samsi K, Campbell S, Abley C, Keady J, Bond J, et al. From forgetfulness to dementia: clinical and commissioning implications of diagnostic experiences. Br J Gen Pract. 2013;63(606):e69–75. 23336476 10.3399/bjgp13X660805 PMC3529295

[8] Organization WH. Global status report on the public health response to dementia: Executive summary. Switzerland: Geneva; 2021.

[9] Neville CE, Young IS, Kee F, Hogg RE, Scott A, Burns F, et al. Northern Ireland Cohort for the Longitudinal Study of Ageing (NICOLA): health assessment protocol, participant profile and patterns of participation. BMC Public Health. 2023;23(1):466. 36899371 10.1186/s12889-023-15355-x PMC9999338

[10] Cadar D, Abell J, Matthews FE, Brayne C, Batty GD, Llewellyn DJ, et al. Cohort Profile Update: The Harmonised Cognitive Assessment Protocol Sub-study of the English Longitudinal Study of Ageing (ELSA-HCAP). Int J Epidemiol. 2020;50(3):725–i6. 10.1093/ije/dyaa227 PMC8271185 33370436

[11] Langa KM, Ryan LH, McCammon RJ, Jones RN, Manly JJ, Levine DA, et al. The Health and Retirement Study Harmonized Cognitive Assessment Protocol Project: Study Design and Methods. Neuroepidemiology. 2020;54(1):64–74. 31563909 10.1159/000503004 PMC6949364

[12] Meng Q, Wang H, Strauss J, Langa KM, Chen X, Wang M, et al. Validation of neuropsychological tests for the China Health and Retirement Longitudinal Study Harmonized Cognitive Assessment Protocol. Int Psychogeriatr. 2019;31(12):1709–19. 31309907 10.1017/S1041610219000693 PMC8082093

[13] Mejia-Arango S, Nevarez R, Michaels-Obregon A, Trejo-Valdivia B, Mendoza-Alvarado LR, Sosa-Ortiz AL, et al. The Mexican Cognitive Aging Ancillary Study (Mex-Cog): Study Design and Methods. Arch Gerontol Geriatr. 2020;91:104210. 32781379 10.1016/j.archger.2020.104210 PMC7854788

[14] Lee J, Khobragade PY, Banerjee J, Chien S, Angrisani M, Perianayagam A, et al. Design and Methodology of the Longitudinal Aging Study in India-Diagnostic Assessment of Dementia (LASI-DAD). J Am Geriatr Soc. 2020;68(Suppl 3):S5–10. 32815602 10.1111/jgs.16737 PMC7503220

[15] Bassil DT, Farrell MT, Wagner RG, Brickman AM, Glymour MM, Langa KM, et al. Cohort Profile Update: Cognition and dementia in the Health and Aging in Africa Longitudinal Study of an INDEPTH community in South Africa (HAALSI dementia). Int J Epidemiol. 2022;51(4):e217–26. 34871405 10.1093/ije/dyab250 PMC9365629

[16] Gross AL, Li C, Briceño EM, Arce Rentería M, Jones RN, Langa KM, et al. Harmonisation of later-life cognitive function across national contexts: results from the Harmonized Cognitive Assessment Protocols. Lancet Healthy Longev. 2023;4(10):e573–83. 37804847 10.1016/S2666-7568(23)00170-8 PMC10637129

[17] Potter C, Feeney J, Fowler E, McKnight AJ, Kee F, McGuinness B. Post-traumatic stress disorder and memory function in older adults exposed to civilian conflict: Findings from the Northern Ireland Cohort for the Longitudinal Study of Ageing (NICOLA). Soc Sci Med. 2025;389:118840. 41317453 10.1016/j.socscimed.2025.118840

[18] McHugh Power JE, Feeney J, Fowler E, McMichael AJ, Hyland P, Lawlor BA, et al. Exposure to the troubles in Northern Ireland, memory functioning, and social activity engagement: results from NICOLA. Eur J Ageing. 2022;19(4):1099–109. 36506685 10.1007/s10433-022-00683-5 PMC9729674

[19] Northern Ireland Audit Office. Mental Health Services in Northern Ireland. Online; 2023.

[20] Kobayashi LC, Jones RN, Briceño EM, Rentería MA, Zhang Y, Meijer E, et al. Cross-national comparisons of later-life cognitive function using data from the Harmonized Cognitive Assessment Protocol (HCAP): considerations and recommended best practices. Alzheimer’s Dement. 2024;20(3):2273–81. 10.1002/alz.13694 PMC10984496 38284801

[21] Feeney J, Monaghan A, McLoughlin S, De Looze C, Oto G, Lawlor B, et al. Cohort Profile Update: The Harmonised Cognitive Assessment Protocol Sub-study of The Irish Longitudinal Study on Ageing (TILDA-HCAP). Int J Epidemiol. 2025;54(1):dyaf008. 10.1093/ije/dyaf008 PMC11821264 39938889

[22] Liu Y, Hayat S, Assaad S, Cadar D, Steptoe A, Lee J et al. Harmonized cognitive assessment protocol in the English longitudinal study of ageing: contrasting approaches to evaluation of factor structure. Int Psychogeriatr. 2025:100042. 10.1016/j.inpsyc.2025.100042 10.1016/j.inpsyc.2025.100042 39915221

[23] Börsch-Supan ADS, Fernández I, Otero MC. Release note to preliminary SHARE-HCAP classification of mild and severe cognitive impairment in five European countries. [Discussion paper]. Munich Center for the Economics of Aging (MEA), SHARE.; 2024.

[24] Jones RN, Manly JJ, Langa KM, Ryan LH, Levine DA, McCammon R, et al. Factor structure of the Harmonized Cognitive Assessment Protocol neuropsychological battery in the Health and Retirement Study. J Int Neuropsychol Soc. 2024;30(1):47–55. 37448351 10.1017/S135561772300019X PMC10787803

[25] Manly JJ, Jones RN, Langa KM, Ryan LH, Levine DA, McCammon R, et al. Estimating the Prevalence of Dementia and Mild Cognitive Impairment in the US: The 2016 Health and Retirement Study Harmonized Cognitive Assessment Protocol Project. JAMA Neurol. 2022;79(12):1242–9. 36279130 10.1001/jamaneurol.2022.3543 PMC9593315

[26] Neville C, Burns F, Cruise S, Scott A, O’Reilly D, Kee F, et al. Cohort Profile: The Northern Ireland Cohort for the Longitudinal Study of Ageing (NICOLA). Int J Epidemiol. 2023;52(4):e211–21. 37011634 10.1093/ije/dyad026 PMC10396407

[27] O’Hara L, Neville C, Marr C, McAlinden M, Kee F, Weir D, et al. Investigating the prevalence of cognitive impairment and dementia in the Northern Ireland Cohort for the Longitudinal Study of Ageing (NICOLA): the Harmonised Cognitive Assessment Protocol (HCAP) cross-sectional substudy. BMJ Open. 2024;14(1):e075672. 38296305 10.1136/bmjopen-2023-075672 PMC10831431

[28] Marr C, O’Hara L, Ward NA, Simoes S, McAlinden M, Sterling C et al. Cohort profile update: the harmonised cognitive assessment protocol sub-study of the Northern Ireland Cohort for the Longitudinal Study of Ageing (NICOLA-HCAP). Int J Epidemiol. 2026;18;55(2):dyag029. 10.1093/ije/dyag029 PMC13016766 41784528

[29] Faculty of Medicine HaLSREC. Faculty Research Ethics Committees Online Queen’s University Belfast. https://www.qub.ac.uk/Research/Governance-ethics-and-integrity/Ethics/FacultyResearchEthicsCommittees.

[30] Sikkes SAM, de Lange-de Klerk ESM, Pijnenburg YAL, Scheltens P, Uitdehaag BMJ. A systematic review of Instrumental activities of daily living scales in dementia: room for improvement. J Neurol Neurosurg Psychiatry. 2009;80(1):7. 10.1136/jnnp.2008.155838 19091706

[31] Jones RN. HCAP22: Harmonized Cognitive Assessment Protocol 2022. GitHub; 2022.

[32] Rosseel Y. lavaan: An R Package for Structural Equation Modeling. J Stat Softw. 2012;48(2):1–36.

[33] van Buuren S, Groothuis-Oudshoorn K. mice: Multivariate Imputation by Chained Equations in R. J Stat Softw. 2011;45(3):1–67.

[34] Harrell FE. rms: regression modeling strategies. 8.1-0 ed: R Package 2023.

[35] Rosseel Y. Missing Values—lavaan Tutorial Online: Department of Data Analysis, Ghent University. https://lavaan.ugent.be/tutorial/est.html#missing-values.

[36] Muthén LKM, Bengt O. Mplus User’s Guide: Chap. 5—examples: confirmatory factor analysis and structural equation modeling. Los Angeles: Muthén & Muthén. https://www.statmodel.com/download/usersguide/Chapter5.pdf.

[37] Lee TS. D. A comparison of full information maximum likelihood and multiple imputation in structural equation modeling with missing data. Am Psychol Assoc Psychol Methods. 2021;26(4):466–85. 10.1037/met0000381 33507765

[38] Collins LM, Schafer JL, Kam C-M. A comparison of inclusive and restrictive strategies in modern missing data procedures. Am Psychol Assoc Psychol Methods. 2001;6(4):330–51. 11778676

[39] Lt H, Bentler PM. Cutoff criteria for fit indexes in covariance structure analysis: Conventional criteria versus new alternatives. Struct Equation Modeling: Multidisciplinary J. 1999;6(1):1–55.

[40] Joseph F, Hair WCB, Babin BJ, Anderson RE. Multivariate data analysis. 7th ed. Prentice-Hall.; 2010.

[41] Cheung GW, Cooper-Thomas HD, Lau RS, Wang LC. Reporting reliability, convergent and discriminant validity with structural equation modeling: A review and best-practice recommendations. Asia Pac J Manage. 2024;41(2):745–83.

[42] Banerjee J, Jain U, Khobragade P, Weerman B, Hu P, Chien S, et al. Methodological considerations in designing and implementing the harmonized diagnostic assessment of dementia for longitudinal aging study in India (LASI-DAD). Biodemography Soc Biol. 2020;65(3):189–213. 32727279 10.1080/19485565.2020.1730156 PMC7398273

[43] Gross AL, Khobragade PY, Meijer E, Saxton JA. Measurement and Structure of Cognition in the Longitudinal Aging Study in India-Diagnostic Assessment of Dementia. J Am Geriatr Soc. 2020;68(Suppl 3):S11–9. 32815599 10.1111/jgs.16738 PMC7513554

[44] Cadar DAJ, Steptoe A. Cognitive impairment and dementia in older English adults: Risk factors and diagnostic algorithms. Banks JaN. In: Steptoe A, Zaninotto P, editors. School of Life and Medical Sciences; Faculty of Population Health Sciences; Institute of Epidemiology and Health. London, UK.: University College London; 2020.

[45] Mohebbi M, Nguyen V, McNeil JJ, Woods RL, Nelson MR, Shah RC, et al. Psychometric properties of a short form of the Center for Epidemiologic Studies Depression (CES-D-10) scale for screening depressive symptoms in healthy community dwelling older adults. Gen Hosp Psychiatry. 2018;51:118–25. 28890280 10.1016/j.genhosppsych.2017.08.002 PMC6178798

[46] Butters MA, Whyte EM, Nebes RD, Begley AE, Dew MA, Mulsant BH, et al. The Nature and Determinants of Neuropsychological Functioning in Late-LifeDepression. Arch Gen Psychiatry. 2004;61(6):587–95. 15184238 10.1001/archpsyc.61.6.587

[47] Dotson VM, Resnick SM, Zonderman AB. Differential association of concurrent, baseline, and average depressive symptoms with cognitive decline in older adults. Am J Geriatr Psychiatry. 2008;16(4):318–30. 18378557 10.1097/JGP.0b013e3181662a9c PMC2405887

[48] Liu J, Chen Y, Xie X, Liu B, Ju Y, Wang M, et al. The percentage of cognitive impairment in patients with major depressive disorder over the course of the depression: A longitudinal study. J Affect Disord. 2023;329:511–8. 36863474 10.1016/j.jad.2023.02.133

[49] Fisher D, Dunn J, Dong H. Distinguishing features of depression in dementia from primary psychiatric disease. Discover Mental Health. 2024;4:3. 38175420 10.1007/s44192-023-00057-y PMC10767128

[50] Office for Health Improvement and Disparities. Dementia profile: prevalence and supporting well topics statistical commentary. March 2025. 2025.

[51] Bacigalupo I, Mayer F, Lacorte E, Di Pucchio A, Marzolini F, Canevelli M, et al. A Systematic Review and Meta-Analysis on the Prevalence of Dementia in Europe: Estimates from the Highest-Quality Studies Adopting the DSM IV Diagnostic Criteria. J Alzheimers Dis. 2018;66(4):1471–81. 30412486 10.3233/JAD-180416 PMC6294583

[52] Moore SE, McEvoy CT, Prior L, Lawton J, Patterson CC, Kee F, et al. Barriers to adopting a Mediterranean diet in Northern European adults at high risk of developing cardiovascular disease. J Hum Nutr Diet. 2018;31(4):451–62. 29159932 10.1111/jhn.12523

[53] Matthews FE, Dening T. Prevalence of dementia in institutional care. Lancet. 2002;360(9328):225–6. 12133659 10.1016/S0140-6736(02)09461-8

[54] Potter C, Marr C, O’Hara L, Neville C, McAlinden M, Kee F, et al. Clinical validation of the Harmonised Cognitive Assessment Protocol (HCAP) in a Northern Irish population. Alzheimer’s Dement. 2025;21(S6):e102313.

