# Supplementary material for: Methodological adaptations for applying the Harmonised Cognitive Assessment Protocol diagnostic algorithm in the Northern Ireland Cohort for the Longitudinal Study of Ageing (NICOLA-HCAP)
Source: BMC Res Notes. 2026 May 2;19:259. doi: 10.1186/s13104-026-07847-x (PMC13281591; doi:10.1186/s13104-026-07847-x)
Supplement: Supplementary file 1 — Supplementary Material 1 [file 13104_2026_7847_MOESM1_ESM.docx]

**Additional Files**

**Additional File 1: Figure 1**


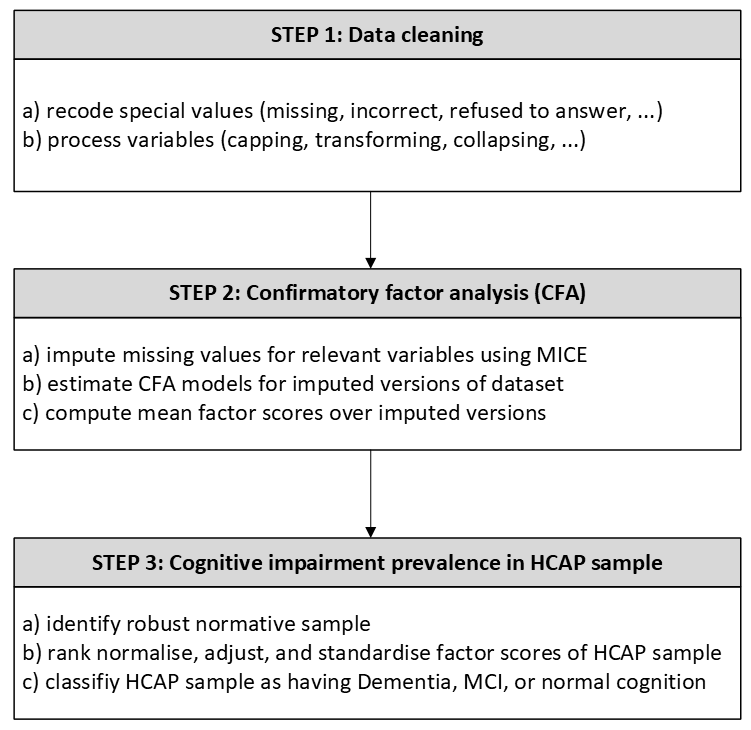


**Figure 1 Overview of the NICOLA-HCAP statistical analysis workflow.**

**Additional File 2**

Data quality checking and monitoring were conducted throughout the study, with monthly data cleaning meetings led by HRS and held in collaboration with TILDA and ELSA teams to ensure harmonised data cleaning procedures.

Data handling procedures included visual inspection of score distributions (e.g., histograms) and descriptive statistical summaries. Binary and categorical variables with sparse response categories (i.e. <5% of responses) were collapsed where appropriate to reduce estimation issues. In total, 10 variables required collapsing due to sparse or skewed response distribution where variables had fewer than 10 distinct response values (e.g., MMSE Orientation, MMSE Recall, MMSE WORLD). These were recoded into binary or ordinal categories to enhance model stability and avoid overfitting to small, unrepresentative subsets of the data(1). Fifteen variables were standardized using z-scores, primarily continuous measures and Trail Making Test Part A and B (both capped at 300) were log-transformed using the formula *1 – log(T)/log(300)*, prior to z-scoring to correct for skewed distributions and ceiling effects in time-based measures. Pairwise correlations were also examined to identify multicollinearity or weak inter-item associations; variables with poor psychometric properties were considered for exclusion. SM1 details the data preprocessing steps used to prepare variables for CFA. Table 1 displays the domain assignment for the variable, description score pre and post processing.

**Table 1: Data handling of neuropsychological assessment listed by cognitive domain, including variable description, and processing steps required prior to confirmatory factor analysis.**

| **Cognitive domain** | **Variable** | **Description** | **Raw score range** | **Processing steps** | **Analytical Variable Range following processing** |
| --- | --- | --- | --- | --- | --- |
| Orientation | MMSE Orientation Score | Sum of correct responses to the ten orientation items (time and place) from the MMSE | 0-10 | Collapse to binary categories:  (0-9) and (10) | 0/1 |
|  | HRS-TICS Name Prime Minister | Whether or not participant was able to correctly name the current Prime Minister of the UK | 0/1 | No change | 0/1 (no change) |
| Memory (immediate) | MMSE Registration | Sum of correctly recalled words from MMSE registration item | 0-3 | Collapse to binary categories:  (0-2) and (3) | 0/1 |
|  | CERAD Word List Recall Immediate Total Score | Sum of correctly recalled words over the 3 immediate recall trials | 0-30 | Standardise to z-score | - |
|  | Brave Man Immediate Gist Score | Sum of item level response for Brave Man immediate recall using gist scoring | 0-12 | Standardise to z-score | - |
|  | Logical Memory Immediate Exact Score | Sum of item level responses for Logical Memory immediate recall using exact scoring | 0-25 | Standardise to z-score | - |
| Memory (delayed) | MMSE Recall | Sum of correctly recalled words from MMSE recall item | 0-3 | 1. collapse to two categories: (0-2) and (3) | 0/1 |
|  | CERAD Word List Recall Delayed Total Score | Sum of correctly recalled words from the delayed recall trial | 0-10 | Standardise to z-score | - |
|  | Brave Man Delayed Gist Score | Sum of item level response for Brave Man delayed recall using gist scoring | 0-12 | Standardise to z-score | - |
|  | Logical Memory Delayed Exact Score | Sum of item level responses for Logical Memory delayed recall using exact scoring | 0-25 | Standardise to z-score | - |
|  | Constructional Praxis Delayed Total Score | Sum of criteria met for constructional praxis delayed recall | 0-11 | Standardise to z-score | - |
| Memory (recognition) | CERAD Word List Recall Recognition Total Score | Sum of correct responses on CERAD word list recognition test | 0-20 | Standardise to z-score | - |
|  | Logical Memory Recognition Total Score | Sum of correct responses on logical memory recognition test | 0-15 | Standardise to z-score | - |
| Visuospatial | MMSE Drawing | Whether or not participant was able to correctly reproduce pentagons from MMSE | 0/1 | Standardise to z-score | - |
|  | Constructional Praxis Immediate Total Score | Sum of criteria met for constructional praxis immediate | 0-11 | Standardise to z-score | - |
| Set shifting | Number series W-Score | Total score calculated according to HRS instructions | 409-584 | Standardise to z-score | - |
|  | Raven’s Matrices Total Score | Sum of correct responses from Raven’s | 0-17 | Standardise to z-score | - |
|  | Trail Making Test Part B Time* | Number of seconds to complete Trails B | 0-300 (capped) | 1. apply log transformation  2. standardise to z-score | - |
| Attention/Speed of Processing | MMSE WORLD | Number of correctly placed letters when spelling WORLD backwards in MMSE | 0-5 | Collapse to binary categories:  (0-4) and (5) | 0/1 |
|  | MMSE Serial 7s | Number of correct backward counts in 7s | 0-5 | Collapse to three categories: (0-2), (3-4), and (5) | 0/1/2 |
|  | Letter Cancellation number correct | Number of correctly crossed out letters from letter cancellation test | 0-65 | Standardise to z-score | - |
|  | Backward Count Total Score | Number of correct backward counts | 0+ | Standardise to z-score | - |
|  | SDMT Total Score | Total number attempted minus number of errors on SDMT | 0-110 | Standardise to z-score | - |
|  | Trail Making Test Part A Time* | Number of seconds to complete Trails A | 0-300 (capped) | 1. apply log transformation  2. standardise to z-score | - |
| Language/Fluency | MMSE Naming Total Score | Number correctly named objects from MMSE | 0-2 | Collapse to binary categories:  (0-1) and (2) | - |
|  | MMSE Reading | Whether or not participant correctly followed instructions on card | 0/1 | No change | - |
|  | MMSE Writing | Whether or not participant wrote proper sentence | 0/1 | No change | 0/1 (no change) |
|  | HRS-TICS Naming Total Score | Number correctly named objects from TICS | 0-2 | Collapse to binary categories:  (0-1) and (2) | 0/1 |
|  | Animal Naming Total Score | Total number of correctly named animals | 0+ | Standardise to z-score | - |
|  | CSID Total Score | Sum of correct responses on CSID items | 0-4 | Collapse to binary categories:  (0-3) and (4) | 0/1 |

**Additional Files 3**

CFA models were estimated in 1,019 NICOLA-HCAP participants after excluding 18 individuals with high missing cognitive data (>25%), to minimize influence of poorly informative patterns affecting covariance and convergence (2). Models were fitted across multiple imputed datasets, with non-converging models removed. CFA was limited to participants with ≤25% missing data, but factor scores were calculated for all 1,037 participants.

**Missing value statistics**

CFA models were estimated across multiple imputed datasets; models that failed to converge were removed. Discarded models did not contribute to the final factor scores, as they were assumed to be unreliable.

**Table 1.** Summary of missing data for cognitive variables (observed) from the HCAP neuropsychological assessment used in the confirmatory factor analysis (CFA).
**Table 1(A)** reports the total number of missing values per cognitive variable.
**Table 1(B)** presents the number of participants with missing data for each variable. These figures informed the choice of multiple imputation procedures (MICE).

**A**

|  | **#** | **%** |
| --- | --- | --- |
| **Cases with missing values** | **185** | **17.84%** |
| **Observed variables with missing values** | **20** | **80.00%** |
| **Total missing values** | **468** | **1.80%** |

**B**

| **# of missing observed variables** | **# of cases** | **% of cases** | **cumulative %** |
| --- | --- | --- | --- |
| **0** | **852** | **82.16%** | **82.16%** |
| **1** | **119** | **11.47%** | **93.63%** |
| **2** | **25** | **2.41%** | **96.04%** |
| **3** | **5** | **0.48%** | **96.52%** |
| **4** | **8** | **0.77%** | **97.29%** |
| **5** | **6** | **0.57%** | **97.87%** |
| **6** | **2** | **0.19%** | **98.07%** |
| **7** | **3** | **0.28%** | **98.36%** |
| **8** | **4** | **0.38%** | **98.74%** |
| **9** | **2** | **0.19%** | **98.93%** |
| **10** | **3** | **0.28%** | **99.22%** |
| **11** | **1** | **0.09%** | **99.32%** |
| **12** | **1** | **0.09%** | **99.42%** |
| **13** | **2** | **0.19%** | **99.61%** |
| **14** | **1** | **0.09%** | **99.71%** |
| **15** | **2** | **0.19%** | **99.90%** |
| **16** | **1** | **0.09%** | **100.00%** |

**Additional Files 4**

NICOLA-HCAP lacked access to Medicare, so we had to adapt the HRS-HCAP normative sample criteria for NICOLA. The normative sample was defined using additional exclusion criteria to reduce contamination from undetected cognitive impairment. First, to try to match the Medicare informed diagnoses, we could only use informant reports of cognitive and neurological impairments, such as stroke, Parkinson's disease, Alzheimer’s disease, and memory problems. Further exclusions were decided prior to analysis and based on:

- **Neuropsychological assessment:** Evidence of cognitive impairment based on performance in cognitive screening tools, MMSE administered during W2 of NICOLA, and MOCA at W1. The decision to use an MMSE cut-off of ≤24 was harmonised with ELSA-HCAP. Interpretable alongside MMSE was the W1 MOCA (approximately 7 years prior to HCAP), although a more stringent cut-off was used(<26) makes it more sensitive to mild cognitive impairment.
- **Functional status:** Presence of any limitation in activities of daily living (ADLs), or two or more limitations in instrumental activities of daily living (IADLs), identified at NICOLA Wave 1, Wave 2, or during the HCAP interview.
- **Symptom progression:** Reports of cognitive or functional decline from informants, supporting the presence and trajectory of symptoms over time. Specifically including CSI-D cognitive activities score ≥5, which is independent of the diagnostic algorithm and captures early or subtle cognitive and functional decline that may not be detected by performance-based measures.

An additional criterion was proposed: exclusion of individuals on dementia-related medications *(search W2 medication for: Donepezil, Rivastigmine, Galantamine, Memantine).* However, due to small cell counts, the DAC did not approve the release of this information.

**Table 1: Summary statistics for participant exclusions applied to the HCAP sample in identifying the normative reference group.**

| **Exclusion criterion** | **# participants meeting criterion** | **# participants meeting only this criterion** |
| --- | --- | --- |
| **Cognitive brief screeners** | | |
| **W2 MMSE ≤24** | 39 | 7 |
| **W1 MOCA <26** | 333 | 205 |
| **Cognitive & neurological (informant reported)** | | |
| **Stroke** | 45 | 9 |
| **Parkinsons Disease** | 15 | 5 |
| **Alzheimer’s Disease** | *<10* | 0 |
| **Memory Problems** | 26 | 4 |
| **Functional Status** | | |
| **W1 ADL Score > 0** | 116 | 14 |
| **W2 ADL Score > 0** | 110 | 18 |
| **W1 IADL Score ≥2** | 47 | 5 |
| **W2_IADL Score ≥2** | 50 | 1 |
| **Informant reported everyday cognitive function (not used in algorithm)** | | |
| **CSI-D – Cognitive Activities ≥5** | 181 | 74 |

^Of the 1,037 participants in the HCAP sample, 472 were included in the normative sample. W1= NICOLA Wave 1; W2= NICOLA Wave 2; ADL= Activities of Daily Living; IADL = Instrumental ADL. <10^

**Rank normalization**

Regression models using restricted cubic splines were employed to rank-normalise (with Blom offset) factor score estimates for each cognitive domain. NICOLA-HCAP preferred software-defined defaults for spline knot placement, unlike HRS-HCAP, which pre-specified placements. Next, these rank-normalised scores were adjusted for age, sex, and education following HRS-HCAP, and additionally, risk of depression using the CES-D short form measure; this was due to the known impact of depression on specific cognitive domains among older adults, underscoring the importance of accounting for depression in cognitive assessments(3). Here, the rank-normalised scores of the normative sample were regressed on the above four factors, yielding expected scores for all combinations of age, sex, education, and depression. The full HCAP sample's scores were then adjusted by subtracting the expected scores and dividing by the standard error of the regression models. This resulted in the normative sample having adjusted scores with a mean of 0, retaining the standard deviation of the rank-normalised scores. Finally, these scores were converted to a t-score distribution by dividing by the standard deviation of the adjusted scores, multiplying by 10, and adding 50; any negative t-scores were clipped to 0.

**Additional Files 5**


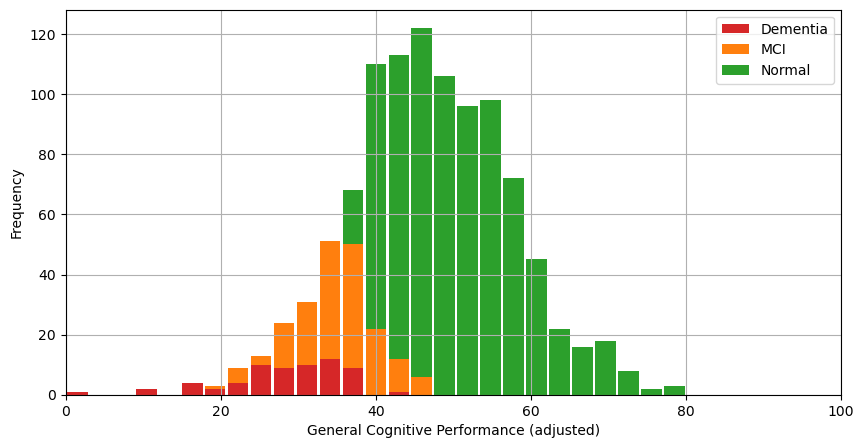


**Figure 1** Distribution of adjusted General Cognitive Performance (GCP) scores across diagnostic groups. Participants are categorised as Dementia (red), Mild Cognitive Impairment (MCI; orange), or Cognitively Normal (green).

1. Harrell FE. Regression Modeling Strategies With Applications to Linear Models, Logistic and Ordinal Regression, and Survival Analysis: Springer Nature; 2015.

2. Enders CK. Applied Missing Data Analysis. 2nd ed: The Guilford Press; 2022.

3. Sweetman J, Stirland LE, Kanaan M, Corley J, Redmond P, Deary IJ, et al. The Relationship Between Anxiety, Depression and Cognitive Functioning in Older Adults: An Exploratory Cross-Sectional Analysis of Wave 1 Lothian Birth Cohort 1936 Data. International Journal of Geriatric Psychiatry. 2024;39(9):e6151.
